# The Multimodal Rehabilitation of Complex Regional Pain Syndrome and Its Contribution to the Improvement of Visual–Spatial Memory, Visual Information-Processing Speed, Mood, and Coping with Pain—A Nonrandomized Controlled Trial

**DOI:** 10.3390/brainsci15070763

**Published:** 2025-07-18

**Authors:** Justyna Wiśniowska, Iana Andreieva, Dominika Robak, Natalia Salata, Beata Tarnacka

**Affiliations:** 1 Department of Rehabilitation, Eleonora Reicher National Institute of Geriatrics, Rheumatology and Rehabilitation, Spartanska 1, 02-637 Warsaw, Poland; justyna.wisniowska@spartanska.pl (J.W.);; 2 Department of Rehabilitation Medicine, Faculty of Medicine, Warsaw Medical University, Spartanska 1, 02-637 Warsaw, Poland; andryana08@gmail.com

**Keywords:** complex regional pain syndrome, pain, visual–spatial memory, information processing speed, depression, coping with pain

## Abstract

**Objectives**: To investigate whether a Multimodal Rehabilitation Program (MRP) affects the change in visual–spatial abilities, especially attention, information-processing speed, visual–spatial learning, the severity of depression, and strategies for coping with pain in Complex Regional Pain Syndrome (CRPS) participants. **Methods**: The study was conducted between October 2021 and February 2023, with a 4-week rehabilitation program that included individual physiotherapy, manual and physical therapy, and psychological intervention such as psychoeducation, relaxation, and Graded Motor Imagery therapy. Twenty participants with CRPS and twenty healthy participants, forming a control group, were enlisted. The study was a 2-arm parallel: a CRPS group with MRP intervention and a healthy control group matched to the CRPS group according to demographic variables. Before and after, the MRP participants in the CRPS group were assessed for visual–spatial learning, attention abilities, severity of depression, and pain-coping strategy. The healthy control group underwent the same assessment without intervention before two measurements. The primary outcome measure was Reproduction on Rey–Osterrieth’s Complex Figure Test assessing visual–spatial learning. **Results**: In the post-test compared to the pre-test, the participants with CRPS obtained a significantly high score in visual–spatial learning (*p* < 0.01) and visual information-processing speed (*p* = 0.01). They made significantly fewer omission mistakes in visual working memory (*p* = 0.01). After the MRP compared to the pre-test, the CRPS participants indicated a decrease in the severity of depression (*p* = 0.04) and used a task-oriented strategy for coping with pain more often than before the rehabilitation program (*p* = 0.02). **Conclusions**: After a 4-week MRP, the following outcomes were obtained: an increase in visual–spatial learning, visual information-processing speed, a decrease in severity of depression, and a change in the pain-coping strategies—which became more adaptive.

## 1. Introduction

Complex Regional Pain Syndrome (CRPS) is a chronic pain condition characterized by prolonged or excessive pain and changes in skin color, temperature, and/or swelling in the affected limb. Unlike neuropathic pain syndromes, which arise from direct nerve injury or disease, CRPS often follows trauma or surgery and involves abnormal inflammatory and sympathetic nervous system responses without a clear nerve lesion [1,2,3]. It is classified under the ICD-10 code G90.5. In clinical examination, patients also mainly perceive allodynia and/or hyperalgesia; sudomotor and vasomotor abnormalities such as swelling, temperature and color changes; and trophic abnormalities can be found as well [3]. The pain is rather regional and does not follow a particular dermatome or myotome. CRPS mainly develops after a trauma, fracture, surgery, or stroke, and it is more common in females [1]. No pathophysiologic mechanism has been discovered for CRPS as yet, and no gold-standard diagnostic test for CRPS exists, but the diagnosis of CRPS is still based on the widely accepted Budapest criteria [4]. The syndrome has a rich historical background, with its earliest descriptions dating back to the 19th century. Paul Sudeck first detailed the post-traumatic bone atrophy now known as Sudeck atrophy, giving the condition its early eponym [5].

CRPS affects approximately 5.5 to 26.2 per 100,000 people per year worldwide, with a higher incidence reported in women, especially between the ages of 40 and 60. The condition is more frequently unilateral, but bilateral cases occur in approximately 8–10% of patients, especially in those with longer disease duration or central sensitization [1,2,4].

The upper extremities—particularly the hands and wrists—are affected more often than the lower limbs. However, CRPS can develop in any limb following trauma or surgery [4].

Diagnosis is primarily clinical, based on the Budapest criteria, but supportive investigations can provide valuable insight [4]. Radiographs in early disease may reveal patchy osteopenia or Sudeck atrophy. MRI can show soft tissue edema, bone-marrow changes, and joint effusions. Bone scintigraphy may demonstrate increased uptake in the affected region [5,6]. Although EMG and nerve conduction studies are typically normal in CRPS-I, they can help exclude other neuropathic disorders or detect nerve damage in CRPS-II [7]. Laboratory tests are generally nonspecific but may be used to rule out infection, rheumatic disease, or other inflammatory conditions [8].

The proper treatment must be induced as soon as possible. The goal of treatment is not only to achieve improvement by lowering the levels of pain and discomfort, but also by functional restoration and prevention of disability. The most optimal management must include complex interventions such as pharmacology, physical and occupational therapies, and behavioral therapy [4]. A Cochrane review from 2022 [9] has shown that among the different physiotherapeutic interventions, mirror therapy and Graded Motor Imagery (GMI) may improve pain as well as function in CRPS. In physical procedures, transcutaneous electrical nerve stimulation, ultrasound, and laser can be used; manual therapy and exercises are also applicable, as well as pain education [10].

Previous studies suggest that patients with the CRPS diagnosis also suffer from neuropsychological symptoms, despite the absence of brain lesions. The neuropsychological disorders best explored in the current literature are changes in body representation, for example, self-reported body representation distribution [7,11], limb laterality recognition problems [11,12,13], and “neglect-like” symptoms associated with disturbances in spatial attention of the CRPS affected side of the body [14,15]. Other cognitive domains such as visual–spatial attention, visual–constructional abilities, and spatial working memory may also be disturbed by the mechanisms of parietal-lobe neural network misconnection problems [7,8,16,17]. A new systematic review and meta-analysis show gray matter abnormalities, especially increased arousal in the left medial superior frontal gyrus and left striatum, and decreased arousal in the corpus callosum, right supplementary motor area, right median cingulate/paracingulate gyri, and right thalamus, based on Voxel-Based Morphometry in patients with CRPS [15]. Chronic pain in CRPS patients could be another reason for the impairment of cognitive functioning, especially attention and working memory capacities [18,19].

Several of the most recent studies have emphasized that chronic neuropathic pain has a modulation effect on mental disorders, especially depression [20,21,22]. Accordingly, CRPS patients are particularly affected by a larger number of depressions compared to normal populations, without chronic neuropathic pain problems [8]. The relationship between depression and neuropathic pain is still unknown, but the main psychological hypothesis concerns emotional reaction to pain and progressive disability [17].

Neuropathic pain can be understood as chronic stress situation [23,24]. The most frequently used stress management and coping strategies are emotion-oriented, avoidance oriented, and task-oriented [25]. The emotion-oriented strategy is understood as focusing on emotional reactions such as fear, anxiety, depression, and embarrassment. The avoidance-oriented strategy focuses on avoiding the resource of stress in thinking, emotional, and behavioral aspects. The task-oriented strategy focuses on problem-solving, time management, and obtaining social support, and thus, it is considered the most beneficial for the patient [26]. The study shows that CRPS patients have a tendency towards pain-related catastrophizing and they are typically used to applying an emotion-oriented strategy, which may be related to lower prefrontal white matter. The lower prefrontal cortex integrity is correlated with a higher degree of catastrophizing in CRPS patients [27].

There are still only a few studies in the current literature describing complex therapies dedicated to CRPS participants. For example, Elomaa et al. [28] showed the effectiveness of a Multimodal Rehabilitation Program (MRP), taking into account movement, pain, and psychological problems in a group of 10 patients with the CRPS diagnosis. The multimodal rehabilitation program was a combination of pharmacotherapy (morphine, memantine), psychological therapy (mindfulness-based, acceptance and commitment therapies), and physiotherapy (individual therapy and GMI) during a 12-week intervention. Motor (hand strength, active ranges of motion, dexterity—health and affected sides), pain, quality of life, depressive symptoms, and pain anxiety symptom variables were measured two times, before and after the intervention. The results showed that after a 12-week multimodal rehabilitation program, compared to the first measurement, a reduction in the pain and CRPS symptoms was obtained; however, no change in psychological indicators was found (quality of life, depressive symptoms, and pain anxiety symptoms). Another short 4-week observational cohort study with 49 CRPS participants was conducted by McCormick et al. [29]. The authors assessed, before and after the rehabilitation program, the severity of depression, pain anxiety, chronic pain acceptance, coping strategies, pain disability, sit-to-stand test, and 6-minute walk test. All patients participated in the multimodal treatment, including group and individual relaxation training assisted by biofeedback, cognitive behavioral therapy and mindfulness-based interventions, occupational therapy (with desensitization techniques, Graded Motor Imagery, and mirror therapy), physical therapy, pool therapy, medical management, and nursing education. After a 4-week intervention, the authors observed an increased usage of the adaptive coping strategy and distraction, and a decreased usage of maladaptive and passive strategies. Patients showed greater chronic pain acceptance and reductions in emotional distress.

Considering the above, we decided to conduct a prospective, interventional, longitudinal control group trial to respond to the interdisciplinary needs of participants with CRPS diagnosis. In accordance with the current literature, it was assumed that the participants with CRPS will present attention disturbances, especially spatial attention, visual–spatial disorders, and problems with visual–spatial learning as a consequence of parietal-lobe neural network misconnection problems [7,8,14,15]. Additionally, participants tend to suffer from depressed moods and use the emotional pain-coping strategy more often than the task-oriented or avoidance-oriented strategy.

The main goal was to check whether a 4-week MRP would impact on attention, visual–spatial abilities, and visual–spatial learning, reduce depression symptoms, and change from an emotion-oriented strategy for coping with pain (catastrophizing and praying or hoping) or avoidance-oriented strategy (diverting attention and increasing behavior activity) to a more adaptive task-oriented strategy (reinterpreting pain sensation, ignoring sensation, and coping self-statements) in patients with CRPS.

## 2. Materials and Methods

### 2.1. Study Protocol

The study involved 2-arm parallel-group (CRPS and control groups) nonrandomized control trials. It was conducted from 10 October 2021 to 1 February 2023. The research protocol was approved by the Ethics Committee (Approval Number: KBT-4/2/2020). The research was completed in accordance with Helsinki Declaration.

The study included a 4-week MRP with psychological, physiotherapeutic, and physical therapy interventions dedicated to CRPS participants. The physical medicine and rehabilitation doctor diagnosed the general condition of the CRPS limb. The study inclusion criteria assumed a clinical diagnosis of CRPS (or equivalent), an age above 18 years old, and a Mini-Mental State Examination (MMSE) score above 24 points. Participants signed an informed consent form prior to the study. Consequently, the exclusion criteria were a non-CRPS diagnosis, an age below 18 years, an MMSE score below 24 points, or patients refusing to consent to their participation in the study. The PhD of psychology (project assistant) assessed the general cognitive functioning by means of MMSE. The Polish adaptation and standardization of MMSE was used to assess general cognitive functioning [30]. The possible score range was from 0 (severe cognitive deficit) to 30 (no cognitive deficit). The threshold at which dementia might be suspected is 24 points.

**Figure 1:**
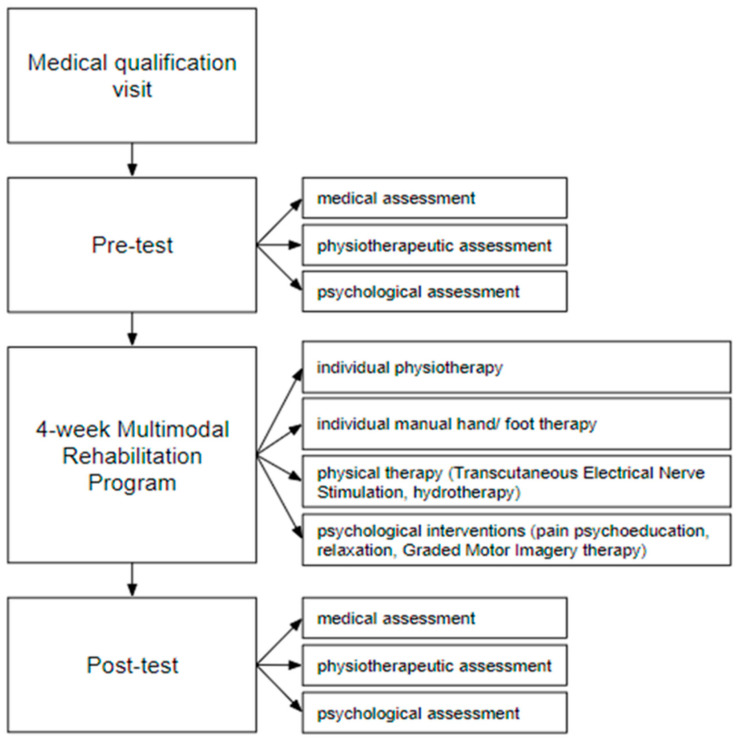
The general scheme of the study.

All participants underwent two medical, psychological, and physiotherapeutic assessments conducted by a rehabilitation medicine specialist (MD), a psychologist (PhD in psychology), and a physiotherapist (MSc in physiotherapy). The Budapest Criteria are a set of clinical criteria used to diagnose CRPS. Possible outcomes include CRPS-I, CRPS-II, CRPS-RSF, or not meeting the Budapest Criteria. The study design is provided in Figure 1.

Pain was measured using the Numeric Rating Scale (NRS). The NRS is a self-reported tool that measures the average pain intensity over the previous 24 h with possible scores ranging from 0 (no pain) to 10 (worst possible pain). The medical assessment included various scales and questionnaires that examined the affected limb condition and central sensitization (Budapest criteria, NRS, central sensitization index). The physiotherapist examination included, in particular, a possible range of motion and muscle strength. The psychological assessment included a series of cognitive-function standardization tests, depression, and pain-coping strategy questionnaires, which were described in the Outcome Measures section. Due to the main study aims, only psychological measures were thoroughly described in the outcome measures section.

The MRP lasted 4 weeks, with sessions 5 times a week (from Monday to Friday), 110 min per day. Every time, the participants underwent the five following procedures: (1) individual physiotherapy (15 min), (2) individual manual hand/foot therapy (15 min), (3) Transcutaneous Electrical Nerve Stimulation (TENS classical, 20 min), (4) hydrotherapy (15 min) and (5) psychological interventions (45 min) as pain psychoeducation, relaxation, and Graded Motor Imagery (GMI) therapy. The psychological and physiotherapeutic 4-week interventional protocol is shown in Table 1.

The individual physiotherapy and physical therapy were conducted by a person holding an MSc in physiotherapy and the psychological intervention was conducted by a person holding an MA in psychology. Therapeutic intervention was conducted by different project assistants than the pre-test and the post-test measurements. All deviations from the study protocol have been included in the appropriate sections.

### 2.2. Physiotherapy and Physical Intervention

Physiotherapeutic intervention included individual physiotherapy, manual hand/foot therapy, and physical therapy, such as hydrotherapy and TENS. Individual physiotherapy (kinesiotherapy) aimed to reduce pain and improving the range of mobility of the affected limb. Main techniques involved fascial therapy, Myofascial Release, joint mobilization, passive and active exercises, and active exercises in water [8,9]. Individual manual hand/foot therapy was carried out using hand/foot devices aiming to improve manual dexterity. Physical therapy included hydrotherapy and TENS, using the thermal and mechanical stimulation of the affected limb. Hydrotherapy was used to decrease pain intensity, reduce swelling, and improve blood circulation [31]. TENS has an analgetic effect, based on Melzack and Wall’s theory of stimulation of nerve fibers [32,33].

### 2.3. Psychological Intervention

Psychological intervention sessions included pain psychoeducation (10 min), relaxation (15 min), and GMI (20 min).

Psychoeducation involved knowledge of the pain mechanism based on the gate control theory and the identification of the factors reducing pain [34]. Other interventions involved stress management and cognitive restructuring according to ABC Ellis’ model [34]. Relaxation was conducted using Shultz autogenic training [35] and Jacobson progressive muscle relaxation [36] with diaphragmatic breathing techniques.

The third element of psychological interventions was Graded Motor Imagery therapy. GMI is a three-step (left/right discrimination, explicit motor imagery, and mirror therapy) program, using a process of imagery movement [37,38,39]. In the left/right discrimination step, participants were presented with a series of photographs and asked to identify as quickly as possible whether the image shows the left or right limb. In an explicit motor imagery step, the participant’s task was to intentionally imagine a movement to replicate the limb’s position shown in the photograph while the affected part of the body remains at rest the whole time. In left/right discrimination and explicit motor imagery steps, The Hand/Foot Recognition App on the tablet was used [40]. The Hand or Foot Module was selected individually, depending on the affected participant’s hand or foot. Stage three involved mirror therapy, which required the patient to place the affected limb inside a special mirror box and observe the movement of their healthy limb in a mirror image. Mirror therapy was conducted using traditional methods with mirror equipment or the Neuroforma software mirror therapy module [41]. The program provides the silhouette from the waist up and an observation of a healthy arm moving in real time on the screen with a mirror image sitting at the front of the screen. However, due to the device’s limitations and the lack of lower-limb module, the Neuroforma’s mirror therapy was conducted only for the upper-limb CRPS participants.

### 2.4. Participants

**Figure 2:**
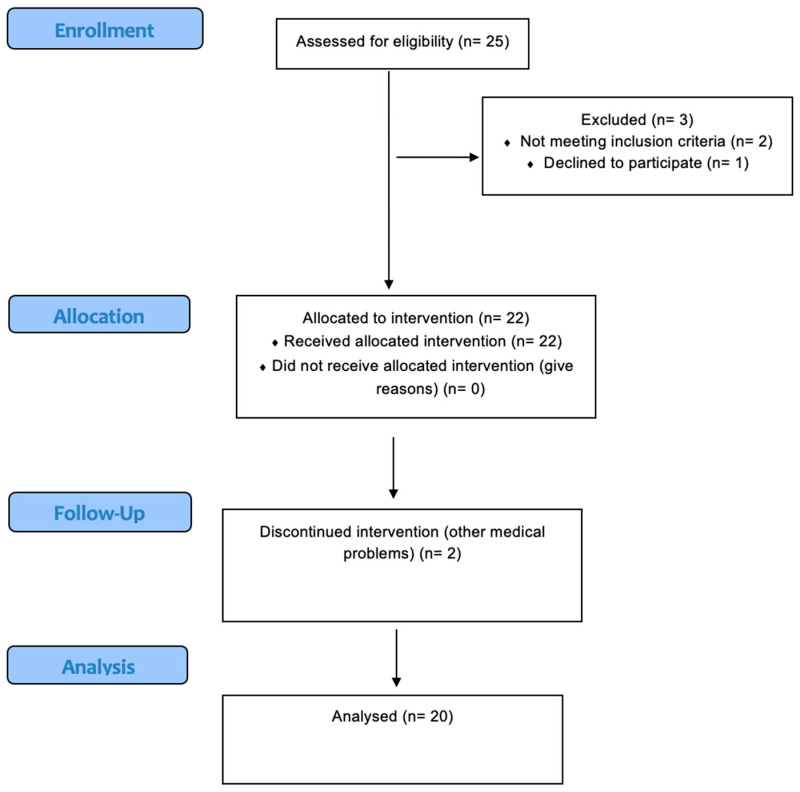
The flow of participants through the study.

Twenty-five participants were recruited from the orthopedic departments in Warsaw and surrounding areas to take part in the study (age 18–73 years old). Fifteen of the participants met the criteria for CRPS-I, three had a diagnosis of CRPS-II, and two were diagnosed with CRPS-RSF. Five participants did not complete the study. The dropouts were due to a suspected stroke (1 person), withdrawal from the psychological part (1 person), a score less than 24 on the MMSE (1 person), a suspected infection (1 person), and non-fulfillment of the CRPS criteria (1 person). During the MRP, the participants were admitted to the stationary rehabilitation department or daily rehabilitation ward based on the doctor’s decision. They did not participate in different kinds of physiotherapy or psychological intervention. The data presented in the study come from the participants who managed to finish the entire study protocol and two measurements (pre-test and post-test). Figure 2 shows the number of participants through each stage of the trial.

Twenty participants (16 females and 4 males) completed the project. The mean age of all the participants was 52.7 (SD = 13.2), the mean number of the years of education was 14.0 (SD = 2.3), the mean of the MMSE score was 28.8 (SD = 1.0), and the mean of the NRS score was 5.0 (SD = 1.9). The general demographic and clinical variables are presented in Table 2.

Thirteen of the participants developed CRPS due to fracture (65%), six as a result of trauma (30%), six were treated surgically followed by immobilization (30%), and twelve were treated conservatively with immobilization (60%). In terms of variation regarding the body areas affected, 15 participants suffered from CRPS of the hand (75%), and for 8 this was the dominant hand (40%), while there were 5 cases of CRPS affecting the leg (25%). The interval between the CRPS diagnosis and the beginning of the MRP ranged from 2 to 96 months (mean 9.9 months). The recruitment of patients to the program occurred most often within a year after the CRPS diagnosis, and written informed consent was obtained from all of the participants. Participants’ demographical and medical details are presented in Table 3.

### 2.5. Control Group

Participants in the control group were matched to the experimental group according to their demographic similarity: gender, age, and number of years of education, with 3 years of acceptable differences (younger or older). The mean age of all the participants was 53.5 (SD = 14.0), the mean number of the years of education was 14.5 (SD = 2.8), and the mean of the MMSE score was 29.4 (SD = 0.1). Pain intensity was not assessed in the control group. There were no demographic differences between experimental and control groups. The MMSE score was statistically different between the CRPS and control groups (*p* = 0.04).

Participants in the control group did not suffer from any chronic musculoskeletal, neurological, cardiological, psychiatric diseases, or chronic pain. The control group did not receive any rehabilitation interventions between the two measurements. The pre-test and post-test measurements were the same as in the experimental group. Participants in the control group, in The Pain-Coping Strategies Questionnaire, were asked to refer to the last pain experience (e.g., toothache, abdominal pain).

### 2.6. Outcome Measures

All primary and secondary outcomes were measured two times: at baseline (pre-test) and after 4 weeks (post-test). An indicator of change for all outcomes was the mean change between two measures (pre-test and post-test).

#### 2.6.1. Primary Outcome

Our primary research question was whether the multimodal rehabilitation could affect the change in visual–spatial abilities and visual–spatial learning in the CRPS participants. This measure was taken from the Reproduction score on Rey–Osterreith’s Complex Figure Test (RCFT) [42]. The RCFT is a task-based test assessing visual–spatial abilities. The Reproduction score measures learning on visual–spatial material after 3 min of recall. Possible scores range from 0 (significant deficit) to 36 (highest possible score). By excluding the learning effect in the second measurement (post-test), the alternative version of RCTF—the Modified Taylor Complex Figure with the same scores range was used [42].

#### 2.6.2. Secondary Outcomes

##### Test of Cognitive Function

Another outcome that could help answer the first research question concerning the possible effect of the change in visual–spatial abilities and visual–spatial learning in the CRPS participants was applying the copy score on the RCFT at 4 weeks [42]. The copy score measures planning on visual material. Possible scores range from 0 (significant deficit) to 36 (highest possible score).

The use of a Benton Visual Retention Test (BVRT) [43] allows for more detailed analyses of visual–spatial working memory and visual–spatial abilities. The BVRT Total Number Correct score counts correct elements on the visual–spatial material. Total Number Correct score counts the correct answers. Possible scores range from 0 (significant deficit) to 10 (highest possible score). The BVRT Number of Errors score counts the number of wrong answers on the visual–spatial material. Possible scores range from 0 (best possible score) to an infinite number of mistakes (worst possible score). This type of error analysis allows us to better understand the problems in visual–spatial working memory. For this purpose, we looked at the types of most common mistakes in BVRT. Possible scores range in all types of mistakes from 0 (best possible score) to an infinite number (worst possible score). The BVRT Distortions error type refers to wrong answers in figure distortion. The BVRT Omissions error type refers to wrong answers in figure omission. The BVRT Misplacements error type refers to incorrect positioning of the figure. The BVRT Perseverations error type refers to incorrect figure repetition. The BVRT Rotations error type refers to wrong answers in figure rotation. The BVRT Size error type refers to changing the figure size incorrectly.

Our secondary research question focused on whether multimodal rehabilitation could affect the change in attention functions in the CRPS participants. This measure was taken from the Attention and Perceptiveness Test—starting version (TUS) [44]. The TUS is a task-based test assessing attention during the 3-min deletion of certain patterns (2 types of stars). The TUS Speed of Work score measures information-processing speed in visual–spatial attention by the number of signs deleted in 3 min (correct and incorrect deletion of signs). Possible scores range from 0 (worst possible score) to 972 (perfect score). The TUS Number of Mistakes score measures distraction resistance in the number of incorrect deletions. Possible scores range from 0 (perfect score) to 972 (worst possible score). The TUS Number of Omissions measures distraction resistance in the number of omissions of correct signs. Possible scores range from 0 (perfect score) to 349 (worst possible score). Other tools that measure attention functions, which we used, were Color-Trials Test part 1 (CTT-1) and Color-Trials Test part 2 (CTT-2) [45] The executive time of CTT-1 measures perceptual field search speed, visual information-processing speed and psychomotor speed in time (seconds). The executive time of CTT-2 assesses shifting attention in time (seconds). Possible scores range in CTT-1 and CTT-2 from 0 s (impossibly fast) to an infinite time (unable to complete).

##### Self-Report Measures

The third research question focuses on whether multimodal rehabilitation affects the change in the severity of depression in the CRPS participants. This measure was taken from the Beck Depression Inventory-II (BDI-II) score at 4 weeks [46]. The BDI-II is a self-reported questionnaire assessing depression severity. Possible scores range from 0 (no depression) to 66 (severe depression).

The last question is associated with the pain-coping strategy and possible changes in the main strategy of coping with pain after the multimodal rehabilitation program. This measure was taken from The Pain-Coping Strategies Questionnaire (CSQ) [47] at 4 weeks. The CSQ is a self-reported instrument assessing different pain-coping strategies. The three main pain-coping strategies measured in this questionnaire are emotion-oriented coping, avoidance-oriented coping, and task-oriented coping scores. The emotion-oriented coping score measures the tendency to have a strong emotional response to pain as a pain-coping strategy. Possible scores range from 0 (strategy not engaged) to 72 (strategy significantly engaged). It is divided into two subscales: Catastrophizing (measures the use of catastrophizing as a pain-coping strategy from 0—strategy not engaged—to 36—strategy significantly engaged-scores) and praying or hoping (measures the use of prayer and wishful thinking as a pain-coping strategy from 0—strategy not engaged—to 36—strategy significantly engaged-scores). The avoidance-oriented coping scale measures the tendency to avoid the source of pain as a pain-coping strategy. Possible scores range from 0 (strategy not engaged) to 72 (strategy significantly engaged). It is divided into two subscales: Diverting attention (measures the use of attention diversion as a pain-coping strategy from 0—strategy not engaged—to 36—strategy significantly engaged) and increased behavioral activity (measures the use of behavioral activity increase as a pain-coping strategy from 0—strategy not engaged—to 36—strategy significantly engaged). The last of the main pain strategies is task-oriented. The task-oriented coping score measures the tendency to address the source of pain as a pain-coping strategy. Possible scores range from 0 (strategy not engaged) to 108 (strategy significantly engaged). It is divided into three subscales: Reinterpreting pain sensations (measures the use of pain-sensation reinterpretation as a pain-coping strategy from 0—strategy not engaged—to 36—strategy significantly engaged-scores), ignoring sensation (measures the use of ignoring pain sensation as a pain-coping strategy from 0—strategy not engaged—to 36—strategy significantly engaged) and coping self statements (measures positive self-affirmations as a pain-coping strategy from 0—strategy not engaged—to 36—strategy significantly engaged).

### 2.7. Research Apparatus

During the physical therapy interventions, a TENS machine (Technomex Multitronic MT-3, Gliwice, Poland) and hydrotherapy for the upper and lower limbs (Technomex, model 1114T and 1117) were used.

The following apparatus was used during the psychological interventions: iPad (8th Generation, 14.8 iPadOS software, Cupertino, CA, USA) with Noigroup’s Hand Recognise and Foot Recognise Apps installed to train the first two stages of GMI (left/right discrimination, explicit motor imagery), Medilab Therapy Mirror Box (size of the box opening: 35 × 24 × 24 cm; size of the acrylic mirror: 31.5 × 21.5 × 1.5 cm, Medilab, Miami, FL, USA) for the third stage of GMI (mirror therapy), with both upper and lower limb-affected patients and a mirror therapy module. The Neuroforma device (Neuroforma MobileBox equipped with a Kinetic video camera, 28-inch screen, and computer program, Neuroforma, Warsaw, Poland) works on the basis of motion capture using a webcam and enables the analysis and correction of a virtual image (augmented reality). The participants were seated in front of the screen (1.5 m away).

### 2.8. Statistical Analyses

The sample size has not been previously estimated using the power analysis. In order to eliminate type II error, post hoc power analyses were conducted using G*power 3.1. For the 0.8 effect size, and with 20 participants in two groups (experimental and control) in the primary outcome with *p* < 0.05, a power size of 0.67 was obtained.

All analyses were carried out using IBM SPSS Statistics 28. At the beginning, the differences among demographic and clinical variables (age, years of education, MMSE score, gender) via one-way repeated measures ANOVA with post hoc Mann–Whitney-U test [48] were calculated.

Secondly, the Shapiro–Wilk test with a significance level *p* < 0.05 [49] corrected for Bonferroni’s multiple comparisons *p* = 0.0125 was used to establish whether the datasets had a normal distribution. The test indicated the lack of normal distribution for the obtained data, so further analysis was carried out using nonparametric tests [50].

In the final part of the analysis, the Wilcoxon (*W*) signed-rank paired test [50], with a significance level *p* < 0.05 and corrected for Bonferroni’s multiple comparisons *p* = 0.0083 [51], was used to compare the pre-test and post-test cognitive functions, mood, and self-ability to cope with pain results. The Spearman rank (r) correlation coefficient [47], with a significance level *p* < 0.05, was used to understand the relationship between the pre-test and the post-test in the CRPS group. The effect-size results were determined and interpreted using the following correlation values: 0.00–0.19 “very weak”; 0.20–0.39 “weak”; 0.40–0.59 “moderate”; 0.60–0.79 “strong”; 0.80–1.0 “very strong” [49].

## 3. Results

As presented in Table 4, the first step in the analysis was to compare the CRPS and control groups’ results obtained on the pre-test and post-test for all primary and secondary outcome measures. The next step was to verify the impact of the MRP on cognitive functions, mood, and self-ability to cope with pain by effect-size measures. The results were nonsignificant (*p* > 0.05).

The results provided in Table 4, in the cognitive-function area, show an increase in visual–spatial learning capacity (RCFT Reproduction, *p* < 0.01) after the Multimodal Rehabilitation Program, in patients with CRPS in the second measurement, compared to the first. The improvement effect of MRP on visual–spatial learning is very strong (r = 0.8, *p* < 0.01). There was no similar interaction between the data in the two measurements in the control group. Furthermore, the group of CRPS patients obtained a decrease in the omission type of errors in the BVRT after the MRP compared to the first measurement (*p* = 0.01) with a moderate effect size of therapy on the correct recall of visual–spatial information from working memory (r = 0.5, *p* = 0.04). No similar results were obtained between the two tests in the control group. Psychomotor speed (execution time of CTT-1, *p* = 0.01) and information-processing speed on visual material (TUS Speed of Work, *p* = 0.01) increased after the MRP compared to the first measurement in the CRPS patients. The impact of MRP on psychomotor speed is very strong (r = 0.8, *p* < 0.01) and the visual information-processing speed is strong (r = 0.7, *p* = 0.01). There were no similar results obtained in the control group between two measurements. Moreover, there were no significant results between the two measurements in the RCFT Copy, executive time of CTT-2, BVRT Total Number Correct, Number of Errors and error type: Distortions, Misplacements, Perseverations, Rotations, Size, TUS Number of Mistakes, and Number of Omissions in the CRPS and control groups. The control group indicated a significant increase in the TUS Number of Mistakes in the second measurement compared to the first, which was not observed in the experimental group.

The results obtained in the mood aspect in the CRPS patients show a reduction in the severity of declared depression symptoms in the second measurement compared to the first (BDI-II, *p* = 0.04), with a strong effect size of the MRP (r = 0.07, *p* < 0.01). There were no significant differences between two measurements in the severity of depression symptoms in the control group.

Regarding strategies for coping with pain, the CRPS group demonstrated an increase in the frequency of use of the task-oriented coping strategy (*p* = 0.02) more often than avoidance and emotional-oriented coping strategies after the Multimodal Rehabilitation Program compared to the first measurement, with a strong effect size of therapy (r = 0.6, *p* = 0.01). Moreover, it was shown that the diverting attention (*p* = 0.01 with strong effect size, r = 0.6, *p* = 0.01) and control over pain (*p* = 0.02 with a strong effect size, r = 0.6, *p* = 0.01) strategies increased after the MRP compared to the first test and the control group results.

The ignoring sensation strategy decreased after the MRP (*p* = 0.01 with a moderate effect size, r = 0.5, *p* = 0.03) compared to the first measurement. There were no significant results in the reinterpreting pain sensation, catastrophizing, praying and hoping, coping, self-statements, and increasing behavioral activity strategies in the CRPS and the control groups between the two measurements. An increased use of catastrophizing as a strategy was more often found in the control group in the second test compared to the patient group results.

Differences between individuals with short-term (≤6 months) and long-term (>6 months) CRPS were assessed. The findings emphasize the cognitive and emotional toll of prolonged CRPS. Long-term CRPS participants reported higher levels of depression (BDI-II) and a greater reliance on emotion-oriented and catastrophizing coping strategies (CSQ) (Table 5).

## 4. Discussion

In this study, we sought to assess the impact of MRP on visual attention, visual–spatial abilities and visual–spatial learning, severity of depression, and strategies for coping with pain in the CRPS participants. We created a prospective, clinical, interventional, and longitudinal control study to answer the research questions about whether the MRP affects the change in visual–spatial abilities, visual–spatial learning, attention functions, visual information-processing speed, severity of depression, and pain-coping strategy. The results of this study indicate an improvement in visual–spatial memory, visual information-processing speed, severity of depression, and changes in strategies for coping with pain after a 4-week Multimodal Rehabilitation Program compared to the healthy control group.

### 4.1. Cognitive Function

To answer the first research question, MRP could improve visual–spatial learning (RCFT Reproduction) in the CRPS participants with a very strong effect size. This result means that participants after a 4-week Multimodal Rehabilitation Program were able to learn more visual–spatial material than before rehabilitation. There was no change between two measurements in visual–spatial material copy (RCFT Copy). In terms of the visual–spatial working memory, we observed an improvement regarding fewer omission mistakes in the BVRT with a moderate effect size. Participants with CRPS rarely missed learning figures after the MRP. Yet, there was no effect in all correctly drawn figures (Total Number Correct in BVRT), number of mistakes in general (BVRT Number of Mistakes) or other mistake types (BVRT Distortions, Misplacements, Perseverations, Rotations, and Size errors). It is possible that these results in the visual–spatial copy and correctness of visual–spatial working memory aspects (BVRT indicators) did not improve because participants with CRPS did not have any difficulty in these cognitive areas before rehabilitation.

Regarding the second research question, participants with CRPS achieved better results after the MRP in two independent indicators measuring visual information-processing speed (executive time of CTT-1 and TUS Speed of Work) with a strong effect size. These results mean that after the MRP, the participants with CRPS could search the visual–spatial field faster than before rehabilitation. There was no impact of the MRP on other attention functions such as shifting (executive time in CTT-2) or distraction resistance (TUS Number of Mistakes and Number of Omissions). It is highly probable that this specific attention function was not affected in the CRPS participants.

It should be noted that in order to execute CTT-1 and TUS, the participants need a functional dominant upper limb to finish the task as quickly as possible (CTT-1) or select as many marks as possible in 3 min (TUS). In our study, 40% of participants had CRPS in their dominant upper limb. This could have contributed to slower execution during the first measurement. These results could mean that there was no improvement in visual processing speed, attention, and the MRP impacting on the dominant hand functions.

In the current literature, the neuropsychological impairments are mostly related to visual–spatial problems associated with the same side of the body as the affected limb, such as visual–spatial perception through bodily sensation, problems with body representation in space, or “neglect-like syndrome” [7,11,14,15,16,17]. There is a lack of literature concerning non-body spatial problems in the CRPS group. Only Libon et al. [17], on a sample of 137 participants, observed impaired working memory span in the CRPS patients. In a study by Kolb et al. [52], the CRPS patients did not experience any difficulties in spatial working memory abilities in a block-tapping test. None of these authors studied visual–spatial learning in CRPS patients or evaluated the effectiveness of any therapeutic programs.

To summarize, the potential mechanisms of improvement after the MRP are understood as an impact of comprehensive rehabilitation in many modalities on the parietal-lobe network [52]. Visual–spatial abilities and attention depend on the proper functioning of parietal lobes, so improvement in these cognitive areas could be a consequence of a more efficient neuron network transfer.

### 4.2. Depressed Mood and Pain-Coping Strategy

In reference to the third research question, after the MRP, we observed a reduction in depression symptoms in Beck Depression Inventory-II with a strong effect size. These results mean that after the rehabilitation program, the participants with CRPS declared fewer depression symptoms compared to the period before rehabilitation. In turn, Elomaa et al. [28] did not observe any changes in severity of depression after a 12-week rehabilitation program. McCormick et al. [29] observed a reduction in emotional distress with increased severity of depression symptoms after a 4-week rehabilitation program with psychological, educational, and physiotherapeutic interventions. Similarly to McCormick et al. [29], we observed mood improvement after the rehabilitation program. Perhaps the mood improvement is observed only in short-time measurements (in a 4-week period), and there were no long-term rehabilitation effects (in a 12-week observation period). In our study, one participant had clinical depression diagnosed by psychiatrists before joining the rehabilitation program. Possibly, the first high score obtained in BDI-II could impact the difference between the two measurements.

Finally, the CRPS participants after the MRP used the task-oriented pain-coping strategy more often than before the intervention, with a strong effect size. As was assumed, the participants used an emotional-oriented pain strategy very often before rehabilitation. They used a task-oriented strategy as well, which we had not expected. The emotional-oriented strategy was also used in the control group, retrospectively associated with the pain sensations. This tendency increased in a 4-week period in comparison to the first measurement. However, after the rehabilitation program, the CRPS group used a task-oriented strategy more often than before. According to the current knowledge, this strategy can help someone cope more constructively in chronic pain situations. The second frequently used strategy after the MRP in our study is the diverted attention strategy, being an avoidance-oriented element strategy. It is assumed that the avoidance-oriented strategy could be beneficial in some chronic stress situations when it is impossible to use the task-oriented strategy. Similarly, McCormick et al. [29] have found that the CRPS participants declared more adaptative pain-coping strategies after a 4-week rehabilitation program as able to mitigate the pain sensation.

In terms of the severity of depression and pain-coping results, it may be assumed that the change in factors after the MRP is due to suitable pain psychoeducation and an increase in self-control when coping with chronic pain. It should be noted that the participants had medium pain intensity (M of NRS = 5.0) when they started the MRP. Perhaps the lower pain intensity allows for greater flexibility and capacity to change the pain-coping strategy.

The presented study has possible applications and shows the need for multimodal rehabilitation, including psychological assessment and interventions in participants with CRPS. This work is one of the few with 20 CRPS participants, which indicates the necessity to combine therapeutic methods in order to help patients facing this problem.

This study has several limitations. First of all, the sample size was small and was not estimated before the start of the study. The rarity and clinical heterogeneity of CRPS posed challenges to recruiting a large and homogeneous patient cohort. As a result, the sample size was relatively small, which may limit the generalizability of our findings. The second limitation is the high heterogeneity of the CRPS clinical variables and the sample demographics indicators, for example, age, comorbidities, or the time between the CRPS diagnosis and the beginning of the rehabilitation program. Another limitation is the lack of an assessment of the effectiveness of mixed psychological and physiotherapeutic interventions. We do not have any details about the effectiveness of single psychological or physiotherapeutic therapies. As with all longitudinal studies with many research assistants, there might be some deviations from the study protocol. For example, we included Neuroforma—assisted mirror therapy—only with the upper-limb module. This means that the participants with lower-limb CRPS had only conventional-based mirror therapy without computer support. Another limitation concerns the sole focus on behavioral data interpretation. It may be difficult to understand the neurobiological mechanism of change before and after the rehabilitation program using neuroimaging techniques.

Further research should be centered around short-term and long-term effectiveness of a multimodal therapeutic program. Perhaps it would be worth investigating the randomized, psychological, or physiotherapeutic separate interventions, combined with or without medication. The neurophysiological bases of rehabilitation programs, as well as the use of neuroimage techniques and neuropsychological tests, should also be included in further works. We recommend caution in extrapolating these results to the broader CRPS population. Future research should aim to address these limitations through larger, multi-center studies with randomized controlled designs to validate and expand upon our findings.

## 5. Conclusions

After a 4-week Multimodal Rehabilitation Program, we observed increasing visual–spatial learning and visual information-processing speed. The severity of depression decreased significantly. Participants with CRPS started using more adaptive strategies to cope with pain, such as a task-oriented strategy.

## Figures and Tables

**Table 1:** The content of physiotherapy and psychological interventions.

Week	Physiotherapeutic Intervention	Psychological Intervention
1	Gentle fascial therapy away from the area of greatest pain, with gradual patient habituation to tactile stimuli within the affected limb.	Introduction to the control gate theory and identification of factors influencing the reduction and increase in pain sensations, introduction to Graded Motor Imagery (GMI) therapy, and first step (left/right discrimination) training.
2	Gradual application of tactile stimuli closer to the painful area and at the painful area itself, anti-edema therapy, neuromuscular therapy.	Psychophysiology of stress and stress management psychoeducation, relaxation practice based on diaphragmatic breathing, second step of GMI (explicit motor imagery).
3	Soft-tissue therapy, muscle relaxation, passive movements, gradual introduction of active movements.	Progressive muscle relaxation practice, third step of GMI (mirror therapy), introduction to Ellis’ ABC model.
4	Joint mobilizations, active movements, active movements with resistance.	Autogenic training relaxation practice, continuation of mirror therapy, introduction to cognitive-restructuring technique.

**Table 2:** Demographic and clinical variables in the CRPS and control groups.

	CRPS Group M (SD)	Control Group M (SD)	*p*-Value ANOVA
Age	52.7 (13.2)	53.5 (14.0)	0.7
Years of education	14.0 (2.3)	14.5 (2.8)	0.7
MMSE	28.8 (1.0)	29.4 (0.1)	0.04
NRS	5.0 (1.9)	-	-
Gender	F—16 M—4	F—16 M—4	0.8

Abbreviations: M—mean; SD—standard deviation; MMSE—Mini-Mental State Examination; NRS—Numeric Rating Scale; F—female; M—men; *p*—significance level.

**Table 3:** Patients’ clinical and demographic details.

N	CRPS Group Age/Sex/Hand/ MMSE/YoE/NRS	Control Group Age/Sex/Hand/ MMSE/YoE	Cause	CRPS Limb	DH	Diagnosis	Period	Comorbidities	Medication
1	73/F/R/30/12/1	71/F/R/30/12	fracture	L hand	N	CRPS I	8	subacromial impingement syndrome	-
2	67/F/R/30/15/5	67/F/R/30/17	fracture	R hand	Y	CRPS I	4	spine osteoarthritis, osteoporosis, diabetes, hyperlipidemia	-
3	45/F/R/28/12/8	48/F/R/28/12	injury	L leg	N	CRPS I	4	-	pregabalin—2 × 150
4	44/M/R/27/17/5	45/M/R/30/18	compound fracture treated with surgery	R hand	Y	CRPS I	3	-	-
5	52/F/R/29/12/5	52/F/R/30/12	injury	R leg	N	CRPS I	3	obesity	-
6	66/F/R/28/14/5	65/F/R/30/12	fatigue fracture	L leg	N	CRPS-RSF	9	-	tramadol, paracetamol—2 × 37.5 + 325 meloxicam—1 × 15
7	37/F/R/29/18/5	38/F/R/29/18	injury	L hand	N	CRPS II	3	-	tramadol, paracetamol—2 × 37.5 + 325
8	57/F/R/30/15/4	59/F/R/29/12	injury	L hand	N	CRPS I	6	-	-
9	64/F/R/29/11/8	62/F/R/30/12	fracture injury treated with surgery	R hand	Y	CRPS I	24	osteoporosis	-
10	52/F/R/29/13/4	50/F/R/27/12	fracture injury	R hand	Y	CRPS I	4	hyperlipidemia	-
11	32/M/R/28/15/1	29/M/R/30/12	injury, incised wound, treated with surgery with immobilization	L hand	N	CRPS II	5	-	-
12	54/F/R/30/14/5	56/F/R/30/16	fracture treated with surgery	L leg	N	CRPS-RSF	8	-	duloxetine—1 × 60
13	68/M/R/28/12/5	70/M/R/27/12	fracture treated with surgery	L hand	N	CRPS I	5	-	-
14	27/F/R/27/11/7.5	25/F/R/30/12	injury, carpal tunnel surgery, radiofrequency ablation	R hand	Y	CRPS I	96 (8 year)	depression, obesity	oxycodone—1 × 240 pregabalin—1 × 150, memantine—1 × 20, quetiapine 1 × 75, duloxetine—1 × 30, propranolol hydrochloride—3 × 10
15	38/M/R/30/15/7	38/M/R/18/17	fracture	R leg	N	CRPS I	2	hypertension	pregabalin—2 × 150
16	69/F/L/28/18/5	69/F/R/29/20	injury with fracture	L hand	Y	CRPS I	3	hyperlipidemia, Sjogren’s syndrome	-
17	63/F/R/29/17/3	62/F/R/29/18	fracture	L hand	N	CRPS I	3	-	gabapentine—1 × 300
18	48/F/R/30/12/7	49/F/R/30/12	carpal tunnel surgery	R hand	Y	CRPS II	2	-	-
19	44/F/R/28/12/5	41/F/R/30/15	fracture	L hand	N	CRPS I	3	diabetes	-
20	53/F/R/29/14/5	50/F/R/30/15	fracture	R hand	Y	CRPS I	3	-	-

Abbreviations: N—number of patients; MMSE—Mini-Mental State Examination; YoE -years of education; DH—dominant hand; F—female; M—Men; NRS—Numeric Rating Scale (0–10); Cause—cause of illness; R—right; L—left; Y—yes; N—no; CRPS limb—CRPS location; CRPS I—CRPS type 1; CRPS II—CRPS type 2; CRPS-RSF—Complex Regional Pain Syndrome Remission of Some Features; Period—time between CRPS diagnosis and start of MRP; Medication—drugs using during MRP.

**Table 4:** The pre-test and post-test comparison results in the CRPS and control groups.

Test	Indicator	Group	Pre-Test M (SD)	Post-Test M (SD)	Pre-/Post-Test W—Wilcoxon *p*-Value	Effect Size *p*-Value
RCFT	Copy	CRPS Control	33.8 (3.3) 34.5 (2.4)	35.3 (1.2) 34.8 (2.3)	82.0 (0.2) 49.5 (0.1)	−0.4 (0.13) 0.6 (0.01)
	Reproduction	CRPS Control	18.38 (6.7) 21.1 (6.2)	23.3 (6.3) 21.1 (5.5)	188.5 (<0.01) 93.0 (0.9)	0.8 (<0.01) 0.9 (<0.01)
BVRT	Total Number Correct	CRPS Control	4.6 (1.5) 8.5 (1.8)	5.3 (1.7) 7.6 (2.2)	77.0 (0.3) 44.0 (0.2)	0.7 (0.02) 0.2 (0.54)
	Number of Errors	CRPS Control	7.5 (2.4) 2.5 (3.2)	6.7 (3.2) 3.1 (3.3)	50.0 (0.1) 75.5 (1.0)	0.7 (0.01) 0.2 (0.38)
	Omissions	CRPS Control	1.1 (1.1) 0.4 (0.9)	0.3 (0.7) 0.5 (1.1)	5.0 (0.01) 12.0 (0.8)	0.5 (0.04) 0.3 (0.34)
CTT	CTT-1	CRPS Control	52.2 (24.7) 48.3 (23.7)	41.6 (13.9) 46.8 (28.6)	35.5 (0.01) 81.5 (0.6)	0.8 (<0.01) 0.7 (<0.01)
	CTT-2	CRPS Control	96.5 (30.4) 91.6 (39.0)	92.5 (30.7) 90.0 (40.1)	77.5 (0.5) 74.5 (0.3)	0.7 (0.01) 0.7 (<0.01)
TUS	Speed of Work	CRPS Control	245.2 (96.9) 346.4 (96.7)	301.1 (86.6) 365.9 (133.7)	176.0 (0.01) 126.0 (0.2)	0.7 (0.01) 0.9 (<0.01)
	Number of Mistakes	CRPS Control	4.3 (6.5) 0.3 (0.6)	3.4 (8.2) 1.5 (2.9)	34.5 (0.3) 33.0 (0.03)	0.3 (0.29) 0.2 (0.41)
	Number of Omission	CRPS Control	9.4 (11.9) 4.1 (3.4)	5.6 (4.9) 4.6 (4.6)	59.5 (0.3) 70.5 (0.6)	0.2 (0.51) 0.6 (0.01)
BDI-II	Total score	CRPS Control	15.3 (9.6) 6.55 (7.0)	10.3 (9.1) 5.6 (7.8)	32.5 (0.04) 31.0 (0.05)	0.7 (<0.01) 0.9 (<0.01)
CSQ	Emotion-oriented Coping	CRPS Control	29.0 (14.2) 20.7 (15.4)	25.1 (11.9) 15.6 (12.4)	58.5 (0.1) 43.5 (0.2)	0.7 (<0.01) 0.8 (<0.01)
	Catastrophizing	CRPS Control	12.5 (9.1) 7.2 (8.3)	9.9 (7.8) 5.0 (6.2)	29.5 (0.08) 16.0 (0.04)	0.8 (<0.01) 0.9 (<0.01)
	Praying or Hoping	CRPS Control	16.7 (7.7) 13.6 (8.5)	15.7 (6.8) 11.5 (8.8)	47.5 (0.5) 26.0 (0.2)	0.7 (<0.01) 0.7 (<0.01)
	Avoidance-oriented Coping	CRPS Control	25.5 (14.0) 23.3 (15.2)	32.3 (14.4) 21.6 (15.8)	148.0 (0.1) 89.0 (0.06)	0.3 (0.18) 0.8 (<0.01)
	Diverting Attention	CRPS Control	9.3 (6.5) 10.2 (9.5)	13.5 (7.8) 10.2 (7.9)	159.5 (0.01) 112.5 (0.8)	0.6 (0.01) 0.8 (<0.01)
	Increasing Behavior Activity	CRPS Control	15.7 (7.6) 13.1 (6.7)	17.3 (6.6) 11.5 (8.9)	111.5 (0.5) 70.0 (0.3)	0.1 (0.71) 0.7 (<0.01)
	Task-oriented Coping	CRPS Control	38.0 (18.3) 33.7 (16.3)	45.1 (15.5) 33.8 (19.8)	167.0 (0.02) 86.5 (1.0)	0.6 (0.01) 0.7 (<0.01)
	Reinterpreting Pain Sensation	CRPS Control	5.1 (6.9) 6.3 (5.7)	7.0 (7.1) 5.7 (6.2)	97.0 (0.1) 43.5 (0.6)	0.5 (0.02) 0.6 (0.01)
	Ignoring Sensation	CRPS Control	14.1 (7.6) 11.5 (7.2)	10.2 (6.6) 11.1 (7.2)	118.0 (0.01) 84.0 (1.0)	0.5 (0.03) 0.8 (<0.01)
	Coping Self Statements	CRPS Control	21.3 (8.1) 17.6 (8.2)	22.6 (5.8) 15.35 (7.8)	108.0 (0.3) 51.5 (0.4)	0.4 (0.05) 0.5 (0.03)

Abbreviations: RCFT—Rey Complex Figure Test; CTT—Color Trails Test; BVRT—Benton Visual Retention Test; TUS—Attention and Perceptiveness Test; BDI-II—Beck’s Depression Inventory—II; CSQ—Coping Strategies Questionnaire; CTT-1—executive time of Color Trails Test part 1; CTT-2—executive time of Color Trails Test part 2; M—mean; SD—standard deviation; *p*—significance level.

**Table 5:** Neuropsychological test outcomes: short-term vs. long-term CRPS.

Test	Measure	Short-Term CRPS (≤6 m) M(SD)	Long-Term CRPS (>6 m) M(SD)
RCFT	Copy	34.3 (3.2)	32.5 (3.5)
	Reproduction	19.8 (6.7)	15.4 (5.4)
BVRT	Total Correct	4.9 (1.4)	3.8 (1.6)
	Errors	7.2 (2.3)	8.1 (2.6)
	Omissions	0.8 (1.0)	1.5 (1.1)
CTT-1	Completion Time	49.1 (22.6)	61.2 (27.3)
CTT-2	Completion Time	91.0 (31.2)	103.7 (28.9)
TUS	Speed of Work	252.4 (84.2)	232.1 (111.7)
	Mistakes	3.1 (5.4)	6.5 (7.3)
	Omissions	7.6 (10.1)	11.3 (13.2)
BDI-II	Depression	13.2 (8.3)	19.0 (10.2)
CSQ	Emotion-Oriented	27.6 (12.8)	31.2 (15.5)
	Catastrophizing	11.4 (8.0)	13.7 (9.9)
	Praying/Hoping	16.1 (7.2)	17.5 (8.2)
	Avoidance	24.7 (13.7)	27.0 (14.3)
	Diverting Attention	9.9 (6.8)	8.3 (6.2)
	Increasing Activity	15.2 (6.9)	16.7 (8.3)
	Task-Oriented	37.2 (17.5)	39.8 (19.0)
	Reinterpreting Pain	5.7 (6.1)	4.2 (7.3)
	Ignoring Sensation	13.1 (7.3)	15.8 (8.1)
	Coping Statements	20.6 (7.4)	22.9 (8.8)

## Data Availability

Data is unavailable due to privacy restrictions.

## Abbreviations

The following abbreviations are used in this manuscript:

CRPS	Complex Regional Pain Syndrome
GMI	Graded Motor Imagery
MRP	Multimodal Rehabilitation Program
MMSE	Mini-Mental State Examination
CRPS I	Complex Regional Pain Syndrome type I
CRPS II	Complex Regional Pain Syndrome type II
CRPS-RSF	Complex Regional Pain Syndrome Remission of Some Features
NRS -	Numeric Rating Scale
TENS	Transcutaneous Electrical Nerve Stimulation
RCFT	Rey–Osterreith’s Complex Figure Test
BVRT	Benton Visual Retention Test
TUS	Attention and Perceptiveness Test
CTT	1—Color-Trials Test part 1
CTT	2—Color-Trials Test part 2
BDI	II—Beck Depression Inventory-II
CSQ	The Pain-Coping Strategies Questionnaire
